# Integrating Genome Sequencing and Untargeted Metabolomics in Monozygotic Twins with a Rare Complex Neurological Disorder

**DOI:** 10.3390/metabo14030152

**Published:** 2024-03-04

**Authors:** Rulan Shaath, Aljazi Al-Maraghi, Haytham Ali, Jehan AlRayahi, Adam D. Kennedy, Karen L. DeBalsi, Sura Hussein, Najwa Elbashir, Sujitha S. Padmajeya, Sasirekha Palaniswamy, Sarah H. Elsea, Ammira A. Akil, Noha A. Yousri, Khalid A. Fakhro

**Affiliations:** 1 College of Health and Life Sciences, Hamad Bin Khalifa University, Doha P.O. Box 34110, Qatar; rshaath@sidra.org; 2 Laboratory of Genomic Medicine-Precision Medicine Program, Sidra Medicine, Doha P.O. Box 26999, Qatar; 3 Neonatal Division, Sidra Medicine, Doha P.O. Box 26999, Qatar; 4 Department of Pediatric Radiology, Sidra Medicine, Doha P.O. Box 26999, Qatar; 5 Metabolon Inc., Morrisville, NC 27560, USA; 6 Precision Medicine of Diabetes Prevention, Department of Population Genomic Medicine and Human Genetics, Sidra Medicine, Doha P.O. Box 26999, Qatar; 7 Department of Molecular and Human Genetics, Baylor College of Medicine, Houston, TX 77030, USA; 8 Department of Genetic Medicine, Weill Cornell Medical College, Doha P.O. Box 24144, Qatar; 9 Computer and Systems Engineering, Faculty of Engineering, Alexandria University, Alexandria 21554, Egypt

**Keywords:** multi-omics approaches, untargeted metabolomics, whole-genome sequencing, rare complex disease

## Abstract

Multi-omics approaches, which integrate genomics, transcriptomics, proteomics, and metabolomics, have emerged as powerful tools in the diagnosis of rare diseases. We used untargeted metabolomics and whole-genome sequencing (WGS) to gain a more comprehensive understanding of a rare disease with a complex presentation affecting female twins from a consanguineous family. The sisters presented with polymicrogyria, a Dandy–Walker malformation, respiratory distress, and multiorgan dysfunctions. Through WGS, we identified two rare homozygous variants in both subjects, a pathogenic variant in *ADGRG1*(p.Arg565Trp) and a novel variant in *CNTNAP1*(p.Glu910Val). These genes have been previously associated with autosomal recessive polymicrogyria and hypomyelinating neuropathy with/without contractures, respectively. The twins exhibited symptoms that overlapped with both of these conditions. The results of the untargeted metabolomics analysis revealed significant metabolic perturbations relating to neurodevelopmental abnormalities, kidney dysfunction, and microbiome. The significant metabolites belong to essential pathways such as lipids and amino acid metabolism. The identification of variants in two genes, combined with the support of metabolic perturbation, demonstrates the rarity and complexity of this phenotype and provides valuable insights into its underlying mechanisms.

## 1. Introduction

Rare genetic disorders collectively affect up to 400 million people worldwide, making them a significant global health concern [1]. Within this realm, rare disorders are heterogeneous and complex, particularly those related to the central nervous system (CNS). Developmental disorders affecting the CNS often manifest in early childhood and can significantly impact cognitive, motor, and social development, thereby profoundly impacting an individual’s quality of life. Given the diversity of neurological disorders, an adequate diagnosis is imperative for ensuring appropriate management and specialized care [2]. However, due to their heterogeneity and complexity, traditional diagnostic approaches, such as a neurological examination and diagnostic testing, may not always be sufficient to accurately diagnose them, especially given that various disorders often share common symptoms. This can result in misdiagnosis and delayed care, which can exacerbate symptoms and increase the burden on patients and their caregivers [2,3].

Some of the commonly used diagnostic approaches for rare genetic diseases in general are exome sequencing (ES) and whole genome sequencing (WGS), which can identify genetic variations that may be responsible for the disease. However, in some cases, ES and WGS may not fully resolve the disease, and complementary omics approaches can be useful in providing a more comprehensive understanding of the disease. Among these complementary omics, metabolomics has emerged as a powerful tool for investigating neurological diseases. Metabolites are intermediates or end products of cellular metabolism and are directly involved in the functional state of cells. Unlike genomics, which provides static information about the genetic variations present from birth, metabolomics captures the dynamic response of a biological system to its environment. In addition, metabolomics can account for phenotypic variability by identifying different metabolic patterns among individuals, which is a dimension that other omics fields often overlook [4]. Therefore, metabolomics offers a comprehensive understanding of diseases by providing insights into ongoing physiological and biochemical processes while also offering a more personalized perspective of disease susceptibility and progression.

We present a multi-omics workup using genome sequencing and untargeted metabolomics to investigate etiology in a set of infant twin sisters with a complex clinical presentation, including polymicrogyria, chronic respiratory distress, and the involvement of multiple affected organs, including the kidneys and heart.

## 2. Case Report

### 2.1. Clinical Description

Subjects were monochorionic, diamniotic female twins born to second-degree consanguineous Iranian parents through an IVF (in vitro fertilization) pregnancy. They were born prematurely at 35 weeks by c-section with birth weights of 2.3 kg for Twin 1 and 2.73 kg for Twin 2. Their APGAR scores were 4 and 8 at 1 and 5 min, respectively. Both patients required neonatal resuscitation with positive pressure ventilation followed by tracheal intubation in the delivery room due to high FiO_2_ requirement, generalized hypotonia, and poor ventilatory effort. The mother had a gravida of 5 parity of 5 with a single positive history of a previously affected sibling from the same set of parents born with dysmorphic features, polydactyly, retrognathia, micrognathia, and rocker bottom feet with normal microarray, who died at 10 days age.

**Figure 1:**
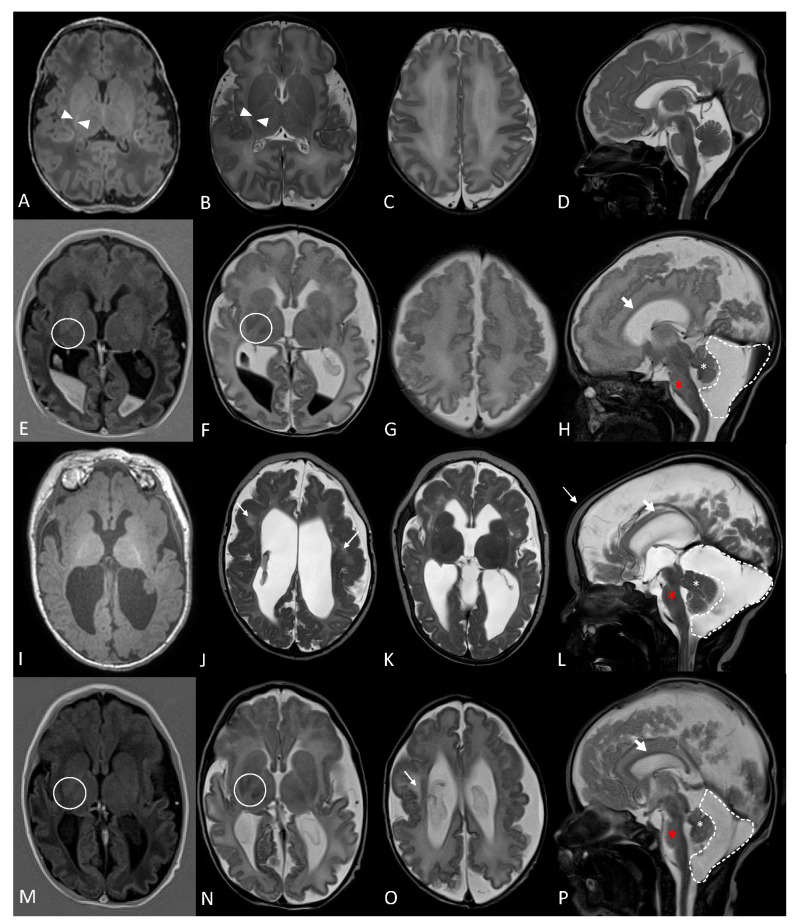
Magnetic resonance imaging of Subjects 1 and 2. (**A**–**D**) Normal MR head performed on an asymptomatic term patient with elevated bilirubin. Axial T1 (**A**), Axial T2 (**B**,**C**), and Sagittal T2 (**D**) images show the expected normal T1 hyperintense, T2 hypointense myelin signal within the posterior limb of the posterior capsule (**A**,**B**, white arrowhead) with a normal gyration pattern and midline structures, including the corpus callosum, brainstem, and cerebellum. (**E**–**H**) Subject 1 at 11 days of age. Axial T1IR (**E**), Axial T2 (**F**,**G**), and Sagittal T2 (**H**) images show the absence of the normal myelin signal within the posterior limb of the internal capsule (**E**,**F**, Circle) and extensive polymicrogyria with frontoparietal predominance. Brain atrophy noted with secondary enlargement of the lateral ventricles. Intraventricular hemorrhage is present. The corpus callosum is thinned (**H**, thick white arrow). The superior cerebellar vermis is dysplastic (**H**, white asterisk), and the pons are hypoplastic (**H**, red asterisk). A large mega cisterna magna is also noted (**H**, dashed line), containing some blood. (**I**–**L**) Subject 1 at 4 months of age. Axial T1 (**I**), Axial T2 (**J**,**K**), and Sagittal T2 (**L**) images show interval progression of myelination and development of white matter changes (**J**,**K**), thin white arrow). (**M**–**P**) Subject 2 at 8 days of age. Axial T1IR (**M**), Axial T2 (**N**,**O**), and Sagittal T2 (**P**) images show the absence of the normal myelin signal within the posterior limb of the internal capsule (**M**,**N**, Circle), extensive polymicrogyria with frontoparietal predominance, and patchy white matter changes. Brain atrophy noted with secondary enlargement of the lateral ventricles. The corpus callosum is thinned (**P**, thick white arrow). The superior cerebellar vermis is dysplastic (**P**, white asterisk), and the brainstem, specifically the pons, is hypoplastic (**P**, red asterisk). A large mega cisterna magna is also noted (**P**, dashed line).

The antenatal history for the twins was remarkable, with multiple congenital abnormalities on the ultrasound present in both twins, including bilateral choroid plexus cysts, retrognathia, nephromegaly with hydronephrosis, and polydactyly. After birth, a detailed neuroimaging assessment was performed for both subjects (as shown in Figure 1). The subjects showed extensive polymicrogyria with frontoparietal predominance with abnormal myelination for chronological age.

Both children had prolonged hospitalization and technology dependence at home for ventilation and feeding. They died before the age of 2 years with acute chronic respiratory failure.

### 2.2. Whole Genome Sequencing Data Analysis

Whole genome sequencing was performed on all available family members, including the twins and their parents. The sibling who passed away 10 days after birth was not included due to sample unavailability. Genomic DNA was extracted from whole blood using the DNeasy Blood & Tissue Kit (QIAGEN, Tokyo, Japan). Subsequently, whole-genome libraries were prepared utilizing the TruSeq DNA Nano kit (Illumina, San Diego, CA, USA). The samples were then subjected to sequencing on the Illumina HiSeq X platform, generating 150 bp length reads with an average sequencing depth of 30×. Data were annotated using our in-house pipeline. Variant prioritization was based on rare variants with a mean allele frequency (MAF) of less than 1% in databases such as gnomAD v2.1.1. Additionally, variants with deleterious predictions according to CADD, PolyPhen, and SIFT were prioritized, as well as variants that demonstrated conservation across multiple species. Given the consanguineous nature of the parents, we further prioritized recessive variants.

The chromosomal microarray results for the twin subjects did not reveal significant abnormalities, and the copy number variation analysis using an in-house pipeline did not identify any potential candidates. Genome sequencing yielded 5,328,257 variants, after which family segregation analysis prioritized 16 rare recessive variants with MAF < 1% and CADD > 10 shared between the two siblings. From these recessive variants, two genetic variants correlated with the phenotype, including one each in *ADGRG1* (p.Arg565Trp) and *CNTNAP1* (p.Glu910Val) (Table 1). Both variants were homozygous in both subjects and inherited from the carrier parents. The variant *ADGRG1*(p.Arg565Trp) was classified as pathogenic in ClinVar (VCV000005831.23) with a CADD score of 29.5. The variant in *CNTNAP1* is novel and not previously reported, with a CADD score of 23.9.

Homozygous or compound heterozygous pathogenic variants in *ADGRG1* cause cortical dysplasia with brain malformations (MIM: 606854). Similarly, homozygous or compound heterozygous pathogenic variants in *CNTNAP1* cause hypomyelinating neuropathy, with or without contractures (MIM: 618186, 616286). The clinical presentations of the subjects were systematically compared to those associated with ADGRG1- and CNTNAP1-related diseases, as outlined in Table 2.

### 2.3. Untargeted Metabolomics Profile

The profile of clinical untargeted metabolomics was performed by Baylor Genetics and Metabolon, Inc., as described [4,5]. Residual plasma (500 µL) was extracted from peripheral blood collected in EDTA-coated tubes during recruitment and frozen at −80 °C. The plasma sample was shipped on dry ice to Baylor Genetics overnight in a frozen condition (−80 °C). We considered clinically relevant biochemicals with z-score levels of <−2 or >+2, typically representing molecules that fall into the <2.5% or >97.5% category, respectively, of the pooled control references consisting of presumably healthy pediatric individuals [5]. 

**Figure 2:**
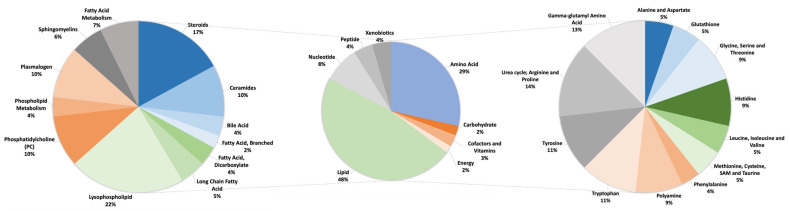
Metabolic perturbations in Subject 1. Middle pie demonstrates a percentage of significant metabolites grouped by their respective biochemical class, with lipid and amino acids contributing 48% and 29%, respectively. The right pie chart shows the sub-pathways of amino acids, and the left pie shows the sub-pathways of lipids. Only sub-classes with at least 2 metabolic perturbations were included, and sub-classes of ceramides, bile acids, steroids, and sphingomyelins were grouped.

An untargeted metabolomic profile was available for Subject 1 only, who was 2 months old at the time of sample collection. Metabolomics analysis revealed 180 clinically relevant biochemicals compared to pooled control references (Table S1). When metabolites fell outside of this physiologic range of abundance, an inborn or acquired defect in metabolism was typically present. Of these metabolites most significantly perturbed in Subject 1, 48% were in lipid pathways (Table S2), followed by 29% in amino acid-related pathways (Table S3) (Figure 2). We observed upregulation in the urea cycle, gamma-glutamyl amino acids, steroids, and lysophospholipids (LPCs), and downregulation in the ceramides, plasmalogens, and phosphatidylcholines, indicating that they may play a role in the pathophysiology of the subject’s condition.

## 3. Discussion

The present study involved twin sisters from a Middle Eastern background with a complex disorder affecting multiple organs, including polymicrogyria and chronic respiratory failure that resulted in their deaths at 19 and 23 months of age. Genetic and metabolic data were utilized to gain a deeper understanding of the molecular biology, physiology, and pathophysiology of this fatal complex disease.

The genetic analysis revealed recessive variants in two genes, *ADGRG1* and *CNTNAP1*. *ADGRG1* encodes a member of the G-protein-coupled receptor family, which is important in the cortical development and physiological functions of the central and peripheral nervous systems, myotube hypertrophy, immune regulation, and myelination development and repair [6]. Pathogenic and likely pathogenic variants in *ADGRG1*, including the established pathogenic p.Arg565Trp variant found in our subjects, led to bilateral frontoparietal polymicrogyria that corresponded with the clinical manifestations of these subjects [7,8,9]. Nonetheless, the subjects presented with additional clinical presentations that were not typical of polymicrogyria.

One of the advantages of WGS over panel testing is the availability of information genome-wide, which allows for the investigation of other possible pathogenic variations shared by the two siblings. In this case, another putatively damaging missense variant was identified in the laminin domain of *CNTNAP1* (c.2729A>T; p.Glu910Val), which contributed to the complexity and severity of the disease. *CNTNAP1* encodes contactin-associated protein 1, a transmembrane cell adhesion protein involved in the proper formation of paranodal axoglial junctions and in the signaling between axons and myelinating glial cells, which are critical for the saltatory conduction of nerve impulses in myelinated nerve fibers [10,11]. More than 30 patients have been reported with the loss of function or missense variants in CNTNAP1 with congenital hypomyelination neuropathy, with or without lethal congenital contracture syndrome [11,12,13,14,15]. In the present case, the subjects had delayed myelination but without contractures. Furthermore, genetic disorders caused by the deficiency of CNTNAP1 and ADGRG1 present similar symptoms [16] (Table 2). However, certain symptoms are specific to the loss of CNTNAP1, such as respiratory distress, retrognathia (Subject 1), low-set ears (Subject 2), absent swallowing, abnormal basal ganglia, and reduced cerebral volume. It is noteworthy that subjects with an ADGRG1-related disease typically survive to adulthood, while subjects with CNTNAP1-related disease die in infancy or early childhood [15,17,18]. The early lethality seen in the twin patients further supports the pathogenicity of the identified *CNTNAP1* variant.

Further differentiating between the two genes are particular MRI findings. MRI findings in patients with the ADGRG1-related disease reveal bilateral polymicrogyria with a frontoparietal predominance, white matter abnormalities, and enlarged ventricles. Brainstem (more specifically pons) hypoplasia is also described, as well as superior cerebellar vermis dysplasia and hypoplasia [19,20]. Variable MRI findings are described in the literature of patients with CNTNAP1-related disease, ranging from normal brain imaging to significant atrophy and white matter changes. The brain MRI findings from previously reported patients included delayed myelination or hypomyelination, cerebral atrophy, and brainstem atrophy [11]. Hengel et al. described similar MRI findings in five patients from two families but, in addition, also described mega cisterna magna in these patients [11]. Subjects 1 and 2 both demonstrated extensive polymicrogyria with a frontoparietal predominance, patchy white matter abnormalities, and a dysplastic superior vermis similar to that described in ADGRG1-related disease (MIM#: 606854, 615752). They both also demonstrated delayed myelination and mega cisterna magna, which are described in patients with CNTNAP1-related disease. Cerebral atrophy, thin corpus callosum, and brainstem atrophy, primarily of the pons, were also noted in both siblings. These latter findings are described in both ADGRG1-related and CNTNAP1-related diseases, though global cerebral atrophy was described more in patients with CNTNAP1-related diseases who had abnormal brain imaging, whereas pontine hypoplasia is only specifically described in ADGRG1-related disease. Therefore, the MRI findings support the contribution of ADGRG1 deficiency, in addition to the deficiency of CNTNAP1 (MIM#: 618186, 616286).

The use of metabolomics in this study provided valuable insights into the pathophysiology of the disease, revealing key perturbations in metabolic pathways and linking them to the subject’s clinical manifestations. For example, in the metabolic profile of Subject 1, there was an accumulation of metabolites in the urea cycle, arginine, and proline metabolism. Accumulation in these metabolites suggests a defect in the urea cycle that causes ammonia and other toxic substances to build up in the blood, which could possibly result in death [21]. Furthermore, the metabolic profile is indicative of oxidative stress, characterized by the disruption of polyamine and glutathione metabolism. This is evident through the accumulation of methionine sulfoxide and polyamines, coupled with a deficiency of cysteinyl molecules [22,23]. Oxidative stress and cellular damage can further exacerbate the impact of the disease [24]. The accumulation of multiple gamma-glutamyl amino acids and 5-oxoproline in the plasma is indicative of a depletion of glutathione, a key antioxidant in the body. This depletion can result in decreased efficiency in the gamma-glutamyl cycle, similar to what is seen in individuals with pyroglutamic acidemia, which is a metabolic disorder characterized by a buildup of pyroglutamic acid in the body [25]. Accumulation of these metabolites is associated with chronic metabolic acidosis, neonatal hemolytic anemia, and variable neurological impairments such as mental impairment and spasticity [25,26]. Remarkably, the patient did have neonatal hemolytic anemia, as signified by the high reticulocyte count and neurological impairment, further suggesting a potential connection between the metabolic profile and the observed phenotype.

An enrichment in the lipid pathways was observed in plasma from Subject 1. Serine is a crucial amino acid involved in several cellular processes, including lipid synthesis, particularly sphingolipid biosynthesis via serine palmitoyltransferase (SPT) enzymes [27]. There were high levels of serine in Subject 1 and low levels of sphingolipids. Moreover, the downregulation of ceramides, as precursors to sphingolipids that regulate cilia formation, coincides with the role of ceramides in the pathophysiology of chronic respiratory distress in subjects in which the cilia structure is impaired [28,29]. Furthermore, other lipids such as phosphatidylcholines, plasmalogens, and LPCs were dysregulated, which is expected, as they are repeatedly reported in neurological and neurodegenerative disease and brain injury [30]. Interestingly, recent studies show how ADGRG1 mediates microglial synaptic refinement via binding to phosphatidylserine, which was found to be reduced in our subject but did not pass the threshold for statistical significance (in the 9th percentile) [31], possibly due to the impaired SPT function. Other lipids, such as LPCs, are also reported to bind to G-coupled protein receptors [32]. ADGRG1 also binds to transglutaminase 2 (TG2), which plays a key role in myelination, suggesting that sphingomyelin and LPCs undergo dysregulation via TG2 [33]. The involvement of ADGRG1 in lipid rafts further supports the phospholipid alterations observed, indicating the potential for lipids in the pathophysiology of disease caused by the established pathogenic variant in *ADGRG1* (p.Arg565Trp) [6].

Moreover, Subject 1 had perturbations in histidine, pyrimidine, purine, tyrosine, and tryptophan metabolism. Patients with dysregulation in these pathways presented with neurological manifestations, including encephalopathy, intellectual disability, developmental delay, and hypotonia, as seen in our subjects [34,35,36,37,38].

Previous studies have revealed communication between the gut microbiota and their associated metabolites with the host’s kidneys and brain through the microbiota–gut–kidney, and gut–brain axes, highlighting the potential influence of the microbiome on the phenotype of the twins [39,40]. Kidney dysfunction was supported by the low levels of carnitine, urate, and creatinine and elevated sedoheptulose (rare metabolite) in Subject 1 [41,42]. Interestingly, we observed a dysregulation in microbiota-associated metabolites, specifically trimethylamine N-oxide and 4-methylcatechol sulfate, which were significantly downregulated, and bile acid was dysregulated [43,44]. It is known that bile acids are one of the most crucial classes of gut microbiota metabolites, as they play key roles in microbiome position, gut metabolism, and cell signaling [45,46]. These findings might indicate a dysfunction in the gut microbiota–kidney and gut–brain axes.

## 4. Conclusions

This study combines high-resolution genetic and metabolic assays to improve our understanding of rare and complex neurodevelopmental disorders. The limitations of this study include the absence of the other twin’s metabolic profile, which could have further supported the observed alterations in metabolic pathways or provided new insights, possibly implicating the role of epigenetic influences. The clinical dilemma that is usually resolved by specific genetic findings was complicated by the discovery of rare homozygous variants in *ADGRG1* and *CNTNAP1* with overlapping neurological symptoms and radiological findings in the same subjects. Given the highly consanguineous nature of the Middle Eastern population, it is not uncommon to discover two genetic causes of disease in the same subject. Therefore, accurate diagnosis with clear pathognomonic findings is important for proper diagnosis and parental genetic counseling, especially for recurrence risk in future pregnancies. The use of metabolomics further provides a comprehensive view of pathophysiology on a molecular level and sheds light on the potential of metabolic abnormalities to distinguish between different diseases.

## Figures and Tables

**Table 1:** Rare genetic variants in twin subjects. Homozygous genetic variants were identified in *ADGRG1* and *CNTNAP1* in relation to the twin’s clinical presentations. The variants are listed along with their respective chromosomal locations, functional annotations, type, identifiers, and association with disease.

Gene	Chromosome	HGVS DNA Reference	HGVS Protein Reference	Variant Type	ClinGen ID	Zygosity	OMIM Disease
ADGRG1	16q21	NM_001145771.3: c.1693C>T	NP_001139243.1: p.Arg565Trp	missense	CA253611	homozygous	AR cortical dysplasia, complex, with other brain malformations 14A (MIM: 606854)
CNTNAP1	17q21.2	NM_003632.3: c.2729A>T	NP_003623.1: p.Glu910Val	missense	CA399649849	homozygous	AR Hypomyelinating neuropathy (MIM: 618186) AR lethal congenital contracture syndrome (MIM: 616286)

**Table 2:** The observed clinical symptoms affecting each organ in the twins are compared to those of ADGRG1 and CNTNAP1-related disorders, suggesting that both genes are involved in the etiology of the observed syndrome.

	*ADGRG1* ^1^	*CNTNAP1* ^1^	Subject 1	Subject 2
**Head and neck**				
Facial diplegia	-	+	-	-
Micrognathia	-	+	retrognathia	NA
Macrocephaly	-	-	+	-
Microcephaly	-	+	-	-
Low-set ears	-	+	-	+
Ophthalmologic abnormalities	+	+	-	-
Dysmorphic features	-	+	-	-
**Respiratory**				
Neonatal respiratory insufficiency	-	+	+	+
**Gastrointestinal**	-	+	+	+
Absent swallow				
Tube feeding	-	+	+	+
**Kidneys**				
Nephromegaly	-	-	+	+ (polycystic)
**Heart**				
Cardiac arrythmias	-	-	+	+
ASD	-	-	+	+
**Skeletal**				
Contractures	-	some	-	-
Clenched hands	-	+	-	-
Foot deformities	-	+	-	-
Polydactyly	-	-	+	+
**Muscle**				
Hypotonia	+	+	+	+
Increased muscle tone/spasticity	+	+	+	+
**Neurologic**				
DD/ID	+	+	+	+
Psychomotor delay	+	+	+	+
Hyperreflexia (CNS)	+	+	+ (clonus)	+ (clonus)
Hypomyelination	Dysmyelination	+	+	+
Seizures	+	some	+	+
Abnormal basal ganglia	-	+	+	+
Brainstem and cerebellar hypoplasia	+	+	+	NA
Reduced cerebral volume	-	+	+	+
Thin corpus callosum	+	+	+	+

^1^ Clinical presentation from OMIM and the literature. DD/ID—developmental delay/intellectual disability, CNS: central nervous system. (+) indicates phenotype is present, (-) not present, (NA) information not available.

## Data Availability

Individual genome data are unavailable due to privacy or ethical restrictions.

## Supplementary Materials

The following supporting information can be downloaded at: https://www.mdpi.com/article/10.3390/metabo14030152/s1. Table S1: Metabolomics analysis revealed 180 clinically relevant biochemicals compared to pooled control references; Table S2: Significant metabolic perturbations in lipid pathways; Table S3: Significant metabolic perturbations in amino acid-related pathways.
